# 2-Naphthyl quinoxalin-2-yl ether

**DOI:** 10.1107/S1600536809007855

**Published:** 2009-03-11

**Authors:** Noor Doha Hassan, Hairul Anuar Tajuddin, Zanariah Abdullah, Seik Weng Ng

**Affiliations:** a Department of Chemistry, University of Malaya, 50603 Kuala Lumpur, Malaysia

## Abstract

In the crystal structure of the title compound, C_18_H_12_N_2_O, the two fused rings are aligned at 64.2 (1)°; the C—O—C angle is 118.73 (12)°.

## Related literature

For the crystal structure of 1-naphthyl quinoxalinyl ether, see: Hassan *et al.* (2009 ▶).
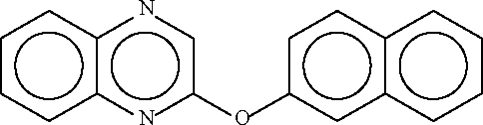


## Experimental

### 

#### Crystal data

C_18_H_12_N_2_O
*M* *_r_* = 272.30
Monoclinic, 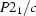

*a* = 6.808 (1) Å
*b* = 7.609 (1) Å
*c* = 26.234 (3) Å
β = 92.522 (2)°
*V* = 1357.5 (3) Å^3^
*Z* = 4
Mo *K*α radiation
μ = 0.08 mm^−1^
*T* = 295 K
0.45 × 0.15 × 0.10 mm

#### Data collection

Bruker SMART APEX diffractometer
Absorption correction: none
7510 measured reflections
3094 independent reflections
1950 reflections with *I* > 2σ(*I*)
*R*_int_ = 0.032

#### Refinement

*R*[*F* ^2^ > 2σ(*F* ^2^)] = 0.048
*wR*(*F* ^2^) = 0.127
*S* = 1.04
3094 reflections
190 parameters
H-atom parameters constrained
Δρ_max_ = 0.17 e Å^−3^
Δρ_min_ = −0.16 e Å^−3^

### 

Data collection: *APEX2* (Bruker, 2007 ▶); cell refinement: *SAINT* (Bruker, 2007 ▶); data reduction: *SAINT*; program(s) used to solve structure: *SHELXS97* (Sheldrick, 2008 ▶); program(s) used to refine structure: *SHELXL97* (Sheldrick, 2008 ▶); molecular graphics: *X-SEED* (Barbour, 2001 ▶); software used to prepare material for publication: *publCIF* (Westrip, 2009 ▶).

## Supplementary Material

Crystal structure: contains datablocks global, I. DOI: 10.1107/S1600536809007855/tk2387sup1.cif

Structure factors: contains datablocks I. DOI: 10.1107/S1600536809007855/tk2387Isup2.hkl

Additional supplementary materials:  crystallographic information; 3D view; checkCIF report

## Figures and Tables

**Fig. 1.**
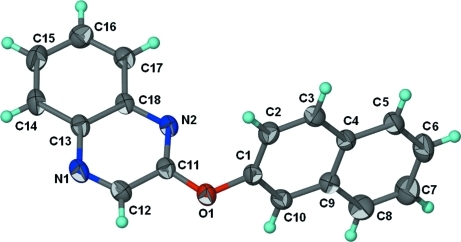
Thermal ellipsoid plot (Barbour, 2001) of the molecule of C18H12N2O at the 50% probability level. Hydrogen atoms are drawn as spheres of arbitrary radius.

## supplementary crystallographic information

### Experimental

2-Naphthol (2.88 g, 20 mmol) was mixed with sodium hydroxide (0.08 g, 20 mmol) in several drops of water. The water was then evaporated. The paste was heated with 2-chloroquinoxaline (3.29 g, 20 mmol) at 423–433 K for 6 h. The product was dissolved in water and the solution extracted with chloroform. The chloroform phase was dried over sodium sulfate; the evaporation of the solvent gave a product that was recrystallized from an ethyl acetate/hexane.

### Refinement

Carbon-bound H-atoms were placed in calculated positions (C—H 0.93 Å) and were included in the refinement in the riding model approximation, with *U*(H) set to 1.2*U*_eq_(C).

### Crystal data

**Table:**

C_18_H_12_N_2_O	*F*(000) = 568
*M**_r_* = 272.30	*D*_x_ = 1.332 Mg m^−^^3^
Monoclinic, *P*2_1_/*c*	Mo *K*α radiation, λ = 0.71073 Å
Hall symbol: -P 2ybc	Cell parameters from 1541 reflections
*a* = 6.808 (1) Å	θ = 2.7–28.2°
*b* = 7.609 (1) Å	µ = 0.08 mm^−^^1^
*c* = 26.234 (3) Å	*T* = 295 K
β = 92.522 (2)°	Block, colorless
*V* = 1357.5 (3) Å^3^	0.45 × 0.15 × 0.10 mm
*Z* = 4	

### Data collection

**Table:**

Bruker SMART APEX diffractometer	1950 reflections with *I* > 2σ(*I*)
Radiation source: fine-focus sealed tube	*R*_int_ = 0.032
graphite	θ_max_ = 27.5°, θ_min_ = 2.8°
ω scans	*h* = −6→8
7510 measured reflections	*k* = −9→8
3094 independent reflections	*l* = −28→34

### Refinement

**Table:**

Refinement on *F*^2^	Primary atom site location: structure-invariant direct methods
Least-squares matrix: full	Secondary atom site location: difference Fourier map
*R*[*F*^2^ > 2σ(*F*^2^)] = 0.048	Hydrogen site location: inferred from neighbouring sites
*wR*(*F*^2^) = 0.127	H-atom parameters constrained
*S* = 1.04	*w* = 1/[σ^2^(*F*_o_^2^) + (0.0544*P*)^2^ + 0.1253*P*] where *P* = (*F*_o_^2^ + 2*F*_c_^2^)/3
3094 reflections	(Δ/σ)_max_ = 0.001
190 parameters	Δρ_max_ = 0.17 e Å^−^^3^
0 restraints	Δρ_min_ = −0.16 e Å^−^^3^

### Fractional atomic coordinates and isotropic or equivalent isotropic displacement parameters (Å^2^)

**Table:**

	*x*	*y*	*z*	*U*_iso_*/*U*_eq_	
O1	0.11303 (15)	0.36756 (17)	0.59259 (4)	0.0477 (3)	
N1	−0.13352 (19)	0.2389 (2)	0.47654 (5)	0.0459 (4)	
N2	0.25074 (18)	0.24104 (18)	0.52218 (5)	0.0379 (3)	
C1	0.2995 (2)	0.3759 (2)	0.61786 (6)	0.0392 (4)	
C2	0.4441 (2)	0.4866 (2)	0.59948 (6)	0.0447 (4)	
H2	0.4210	0.5499	0.5695	0.054*	
C3	0.6192 (2)	0.5003 (2)	0.62616 (6)	0.0435 (4)	
H3	0.7168	0.5723	0.6138	0.052*	
C4	0.6559 (2)	0.4077 (2)	0.67216 (6)	0.0371 (4)	
C5	0.5053 (2)	0.3003 (2)	0.69082 (6)	0.0394 (4)	
C6	0.3253 (2)	0.2858 (2)	0.66207 (6)	0.0412 (4)	
H6	0.2253	0.2147	0.6735	0.049*	
C7	0.8364 (2)	0.4201 (3)	0.70077 (6)	0.0483 (5)	
H7	0.9372	0.4892	0.6888	0.058*	
C8	0.8642 (3)	0.3322 (3)	0.74553 (7)	0.0618 (6)	
H8	0.9839	0.3414	0.7638	0.074*	
C9	0.7154 (3)	0.2285 (3)	0.76428 (7)	0.0694 (6)	
H9	0.7355	0.1704	0.7953	0.083*	
C10	0.5402 (3)	0.2116 (3)	0.73751 (7)	0.0593 (5)	
H10	0.4424	0.1406	0.7502	0.071*	
C11	0.0985 (2)	0.3003 (2)	0.54445 (6)	0.0375 (4)	
C12	−0.0954 (2)	0.2999 (2)	0.52209 (6)	0.0439 (4)	
H12	−0.1976	0.3445	0.5406	0.053*	
C13	0.0233 (2)	0.1738 (2)	0.45104 (6)	0.0389 (4)	
C14	−0.0076 (3)	0.1015 (3)	0.40208 (6)	0.0520 (5)	
H14	−0.1333	0.1008	0.3867	0.062*	
C15	0.1453 (3)	0.0325 (3)	0.37707 (7)	0.0561 (5)	
H15	0.1237	−0.0157	0.3447	0.067*	
C16	0.3351 (3)	0.0340 (2)	0.39983 (7)	0.0524 (5)	
H16	0.4385	−0.0139	0.3824	0.063*	
C17	0.3705 (2)	0.1044 (2)	0.44705 (6)	0.0430 (4)	
H17	0.4977	0.1063	0.4615	0.052*	
C18	0.2149 (2)	0.1739 (2)	0.47388 (5)	0.0348 (4)	

### Atomic displacement parameters (Å^2^)

**Table:**

	*U*^11^	*U*^22^	*U*^33^	*U*^12^	*U*^13^	*U*^23^
O1	0.0342 (6)	0.0636 (9)	0.0449 (6)	0.0032 (5)	−0.0035 (5)	−0.0096 (6)
N1	0.0344 (8)	0.0508 (10)	0.0517 (8)	−0.0015 (6)	−0.0080 (6)	0.0037 (7)
N2	0.0336 (7)	0.0387 (8)	0.0409 (7)	−0.0003 (6)	−0.0048 (5)	0.0016 (6)
C1	0.0348 (9)	0.0424 (11)	0.0402 (9)	0.0010 (7)	−0.0013 (7)	−0.0076 (7)
C2	0.0497 (10)	0.0453 (11)	0.0389 (9)	−0.0025 (8)	−0.0017 (7)	0.0046 (8)
C3	0.0441 (9)	0.0424 (11)	0.0441 (9)	−0.0095 (8)	0.0033 (7)	0.0009 (8)
C4	0.0376 (8)	0.0339 (10)	0.0398 (8)	0.0010 (7)	0.0004 (6)	−0.0055 (7)
C5	0.0420 (9)	0.0356 (10)	0.0404 (9)	0.0010 (7)	0.0010 (7)	0.0008 (7)
C6	0.0392 (9)	0.0390 (11)	0.0457 (9)	−0.0056 (7)	0.0052 (7)	−0.0005 (8)
C7	0.0382 (9)	0.0534 (13)	0.0529 (10)	−0.0002 (8)	−0.0015 (7)	−0.0099 (9)
C8	0.0510 (12)	0.0723 (16)	0.0603 (12)	0.0077 (10)	−0.0177 (9)	−0.0017 (10)
C9	0.0681 (15)	0.0816 (17)	0.0571 (12)	0.0052 (12)	−0.0130 (10)	0.0210 (11)
C10	0.0594 (12)	0.0617 (15)	0.0566 (11)	−0.0039 (10)	−0.0004 (9)	0.0194 (10)
C11	0.0366 (9)	0.0351 (10)	0.0404 (8)	−0.0013 (7)	−0.0033 (7)	0.0026 (7)
C12	0.0332 (8)	0.0476 (11)	0.0506 (10)	0.0030 (8)	−0.0019 (7)	0.0007 (8)
C13	0.0387 (9)	0.0341 (10)	0.0431 (9)	−0.0050 (7)	−0.0058 (7)	0.0045 (7)
C14	0.0502 (11)	0.0557 (13)	0.0486 (10)	−0.0079 (9)	−0.0138 (8)	−0.0009 (9)
C15	0.0689 (13)	0.0558 (14)	0.0431 (10)	−0.0075 (10)	−0.0046 (9)	−0.0087 (9)
C16	0.0559 (11)	0.0509 (13)	0.0507 (10)	0.0038 (9)	0.0047 (8)	−0.0040 (9)
C17	0.0401 (9)	0.0415 (11)	0.0473 (9)	0.0018 (7)	−0.0013 (7)	0.0025 (8)
C18	0.0360 (8)	0.0295 (9)	0.0386 (8)	−0.0029 (7)	−0.0036 (6)	0.0060 (7)

### Geometric parameters (Å, °)

**Table:**

O1—C11	1.3622 (18)	C7—H7	0.9300
O1—C1	1.4073 (18)	C8—C9	1.391 (3)
N1—C12	1.297 (2)	C8—H8	0.9300
N1—C13	1.377 (2)	C9—C10	1.363 (3)
N2—C11	1.2928 (19)	C9—H9	0.9300
N2—C18	1.3778 (19)	C10—H10	0.9300
C1—C6	1.352 (2)	C11—C12	1.421 (2)
C1—C2	1.398 (2)	C12—H12	0.9300
C2—C3	1.359 (2)	C13—C14	1.405 (2)
C2—H2	0.9300	C13—C18	1.411 (2)
C3—C4	1.410 (2)	C14—C15	1.360 (3)
C3—H3	0.9300	C14—H14	0.9300
C4—C5	1.415 (2)	C15—C16	1.399 (2)
C4—C7	1.415 (2)	C15—H15	0.9300
C5—C10	1.410 (2)	C16—C17	1.361 (2)
C5—C6	1.414 (2)	C16—H16	0.9300
C6—H6	0.9300	C17—C18	1.401 (2)
C7—C8	1.357 (2)	C17—H17	0.9300
			
C11—O1—C1	118.73 (12)	C8—C9—H9	119.8
C12—N1—C13	116.62 (13)	C9—C10—C5	120.83 (19)
C11—N2—C18	115.58 (13)	C9—C10—H10	119.6
C6—C1—C2	122.30 (15)	C5—C10—H10	119.6
C6—C1—O1	117.50 (15)	N2—C11—O1	121.55 (13)
C2—C1—O1	119.95 (14)	N2—C11—C12	124.14 (15)
C3—C2—C1	118.92 (15)	O1—C11—C12	114.31 (14)
C3—C2—H2	120.5	N1—C12—C11	121.72 (15)
C1—C2—H2	120.5	N1—C12—H12	119.1
C2—C3—C4	121.39 (16)	C11—C12—H12	119.1
C2—C3—H3	119.3	N1—C13—C14	119.82 (15)
C4—C3—H3	119.3	N1—C13—C18	121.02 (14)
C3—C4—C5	118.72 (14)	C14—C13—C18	119.14 (15)
C3—C4—C7	122.55 (16)	C15—C14—C13	120.36 (16)
C5—C4—C7	118.72 (15)	C15—C14—H14	119.8
C10—C5—C4	118.70 (15)	C13—C14—H14	119.8
C10—C5—C6	122.33 (16)	C14—C15—C16	120.19 (16)
C4—C5—C6	118.97 (14)	C14—C15—H15	119.9
C1—C6—C5	119.65 (15)	C16—C15—H15	119.9
C1—C6—H6	120.2	C17—C16—C15	120.98 (17)
C5—C6—H6	120.2	C17—C16—H16	119.5
C8—C7—C4	120.72 (17)	C15—C16—H16	119.5
C8—C7—H7	119.6	C16—C17—C18	119.89 (16)
C4—C7—H7	119.6	C16—C17—H17	120.1
C7—C8—C9	120.65 (17)	C18—C17—H17	120.1
C7—C8—H8	119.7	N2—C18—C17	119.67 (14)
C9—C8—H8	119.7	N2—C18—C13	120.91 (14)
C10—C9—C8	120.38 (18)	C17—C18—C13	119.43 (14)
C10—C9—H9	119.8		
			
C11—O1—C1—C6	−119.60 (16)	C18—N2—C11—O1	178.93 (14)
C11—O1—C1—C2	65.9 (2)	C18—N2—C11—C12	−0.3 (2)
C6—C1—C2—C3	2.0 (3)	C1—O1—C11—N2	1.5 (2)
O1—C1—C2—C3	176.20 (15)	C1—O1—C11—C12	−179.20 (15)
C1—C2—C3—C4	−1.1 (3)	C13—N1—C12—C11	0.3 (2)
C2—C3—C4—C5	−0.7 (2)	N2—C11—C12—N1	−0.2 (3)
C2—C3—C4—C7	−179.77 (16)	O1—C11—C12—N1	−179.53 (15)
C3—C4—C5—C10	−178.29 (16)	C12—N1—C13—C14	178.37 (16)
C7—C4—C5—C10	0.8 (2)	C12—N1—C13—C18	0.1 (2)
C3—C4—C5—C6	1.7 (2)	N1—C13—C14—C15	−178.25 (17)
C7—C4—C5—C6	−179.25 (15)	C18—C13—C14—C15	0.0 (3)
C2—C1—C6—C5	−1.1 (3)	C13—C14—C15—C16	−0.4 (3)
O1—C1—C6—C5	−175.37 (14)	C14—C15—C16—C17	−0.2 (3)
C10—C5—C6—C1	179.15 (16)	C15—C16—C17—C18	1.1 (3)
C4—C5—C6—C1	−0.8 (2)	C11—N2—C18—C17	−179.07 (14)
C3—C4—C7—C8	178.35 (17)	C11—N2—C18—C13	0.8 (2)
C5—C4—C7—C8	−0.7 (3)	C16—C17—C18—N2	178.38 (15)
C4—C7—C8—C9	−0.2 (3)	C16—C17—C18—C13	−1.5 (2)
C7—C8—C9—C10	1.0 (3)	N1—C13—C18—N2	−0.7 (2)
C8—C9—C10—C5	−0.9 (3)	C14—C13—C18—N2	−178.94 (15)
C4—C5—C10—C9	0.0 (3)	N1—C13—C18—C17	179.13 (15)
C6—C5—C10—C9	−179.97 (18)	C14—C13—C18—C17	0.9 (2)
